# MAPK activation and *HRAS* mutation identified in pituitary spindle cell oncocytoma

**DOI:** 10.18632/oncotarget.9244

**Published:** 2016-05-09

**Authors:** Michael B. Miller, Wenya Linda Bi, Lori A. Ramkissoon, Yun Jee Kang, Malak Abedalthagafi, David S. Knoff, Pankaj K. Agarwalla, Patrick Y. Wen, David A. Reardon, Brian M. Alexander, Edward R. Laws, Ian F. Dunn, Rameen Beroukhim, Keith L. Ligon, Shakti H. Ramkissoon

**Affiliations:** ^1^ Department of Pathology, Brigham and Women's Hospital, Boston, MA, USA; ^2^ Department of Neurosurgery, Brigham and Women's Hospital, Boston, MA, USA; ^3^ Department of Cancer Biology, Dana-Farber Cancer Institute, Boston, MA, USA; ^4^ Department of Medical Oncology, Dana-Farber Cancer Institute, Boston, MA, USA; ^5^ Department of Neurosurgery, Massachusetts General Hospital, Boston, MA, USA; ^6^ Department of Radiation Oncology, Brigham and Women's Hospital, Boston, MA, USA; ^7^ Department of Radiation Oncology, Dana-Farber Cancer Institute, Boston, MA, USA; ^8^ Broad Institute of MIT and Harvard, Cambridge, MA, USA; ^9^ Department of Pathology, Boston Children's Hospital, Boston, MA, USA; ^10^ Harvard Medical School, Boston, MA, USA

**Keywords:** spindle cell oncocytoma, pituitary, MAPK, HRAS, genomics

## Abstract

Pituitary spindle cell oncocytoma (SCO) is an uncommon primary pituitary neoplasm that presents with mass effect on adjacent neurovascular structures, similar to non-hormone-producing pituitary adenomas. To determine the molecular etiology of SCO, we performed exome sequencing on four SCO cases, with matched normal controls, to assess somatic mutations and copy number alterations. Our analysis revealed a low mutation rate and a copy-neutral profile, consistent with the low-grade nature of this tumor. However, we identified a co-occurring somatic *HRAS* (p.Q61R) activating point mutation and *MEN1* frameshift mutation (p.L117fs) present in a primary and recurrent tumor from one patient. Other SCOs demonstrated mutations in *SND1* and *FAT1*, which are associated with MAPK pathway activation. Immunohistochemistry across the SCO cohort demonstrated robust MAPK activity in all cases (n=4), as evidenced by strong phospho-ERK staining, while phospho-AKT levels suggested only basal levels of PI3K pathway activation. Taken together, this identifies the MAPK signaling pathway as a novel therapeutic target for spindle cell oncocytoma, which may offer a powerful adjunct for aggressive tumors refractory to surgical resection.

## INTRODUCTION

Spindle cell oncocytoma (SCO) is a rare non-endocrine neoplasm of the hypophysis, which exhibits WHO grade I histology [1]. SCO presents similarly to non-functioning pituitary adenomas, clinically demonstrating pituitary hypofunction, visual field deficits, and potential headache and nausea, due to mass effect. Although they were initially regarded as benign, several subsequent recurrent and locally aggressive SCO cases have been reported [2–6]. SCO is primarily treated with surgical resection, while radiation therapy has been reported for patients with recurrence [2]. Invasion of nearby structures, including the cavernous sinus, suprasellar space, and sphenoid sinus, can challenge traditional therapeutic strategies.

Histologically, SCOs show an interlacing fascicular pattern of spindled to epithelioid cells, with eosinophilic and variably oncocytic cytoplasm. Nuclear atypia is generally minimal and mitotic indices are low [2]. Ultrastructural features of abundant mitochondria and a paucity of secretory granules help distinguish SCOs from non-functioning adenomas [1, 2]. Expression of S-100, vimentin, galectin-3, and epithelial membrane antigen (EMA) is typical of SCOs, which lack expression of pituitary adenoma markers such as synaptophysin, chromogranin, and pituitary hormones [1]. SCOs generally do not express glial fibrillary acidic protein (GFAP), distinguishing them from pituicytoma, a tumor derived from neurohypophyseal glial pituicytes [1, 2, 7]. SCOs also do not express cytokeratins, smooth muscle actin (SMA), CD34, or CD68.

The cell of origin for SCOs remains unclear. They have been postulated to derive from folliculostellate cells of the adenohypophysis, based on shared expression of S-100, vimentin, galectin-3, and EMA, as well as desmosomes and intermediate junctions found using electron microscopy [1]. However, the pituicyte has also been proposed as a potential cell of origin, on the basis of shared expression of thyroid transcription factor 1 (TTF-1), prompting a potential classification of SCOs as oncocytic pituicytomas [8].

Little is known about the genetic drivers of proliferation and infiltration in SCO. A recent report on seven cases found no *BRAF* V600E mutations, *BRAF-KIAA* fusions, or *IDH* R132H mutation-specific immunoreactivity [8]. One case report observed mild-to-moderate expression of phospho-AKT, phospho-mTOR, and GLI2, suggesting some degree of activation of mammalian target of rapamycin (mTOR) and sonic hedgehog (SHH) pathways [9].

In order to further examine the molecular drivers of oncogenesis in spindle cell oncocytoma, we performed whole exome sequencing and signal pathway profiling on four cases of SCO. Here we report novel genetic mutations that may provide additional insights into the future treatment of this disease.

## RESULTS

### Mutational profile of SCO

**Figure 1:**
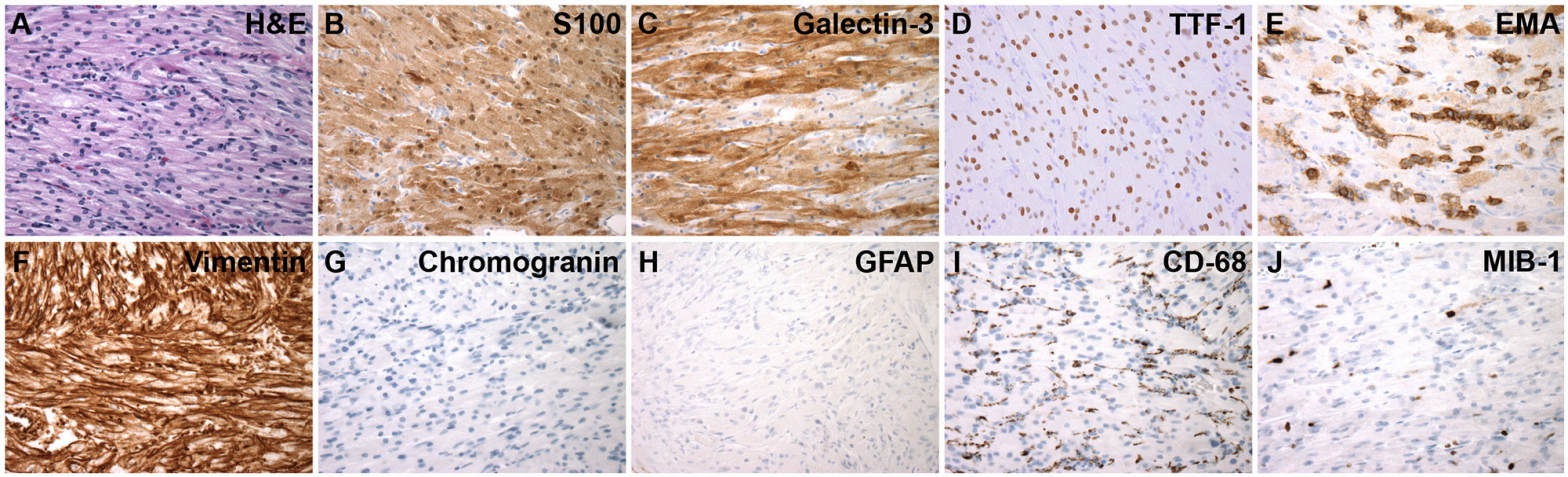
Histologic and Immunohistochemical Features of Spindle Cell Oncocytoma **A.** H&E stain. Immunohistochemistry for: **B.** S100, **C.** Galectin-3, **D.** TTF-1, **E.** EMA, **F.** Vimentin, **G.** Chromogranin, **H.** GFAP, **I.** CD68 and **J.** MIB-1 (Ki-67). (A-J, 600X magnification; A-I, case 1; J, case 2)

We identified all cases of SCO resected at Brigham and Women's Hospital since its first report at this institution in 2002, yielding four cases from three patients (Table 1). Patient three manifested with recurrent/residual tumor less than a year after initial resection, and therefore two separate samples were available for study (cases 3A and 3B). Each SCO case was reviewed and the diagnosis confirmed on the basis of histologic appearance and immunohistochemical profile (Table 2). Figure 1 illustrates typical histologic and immunohistochemical features. In concordance with a recent report [8], we found strong nuclear TTF-1 expression in each case of SCO.

**Table 1:** Clinical profiles of spindle cell oncocytoma cases

Case	Age (Yr)	Imaging characteristics
1	66	2.4 cm sellar mass, abutting cavernous sinuses and third ventricle, and extending into sphenoid sinus and posterior to optic chiasm
2	50	1.4 cm sellar mass, partially surrounding bilateral internal carotid arteries and abutting optic nerves
3A	63	2.7 cm sellar mass, extending into sphenoid sinus, partially encasing bilateral internal carotid arteries, and displacing optic chiasm and optic nerves
3B	63	1.7 cm residual/recurrent enhancing sellar mass, partially encasing left internal carotid artery, with displacement of optic chiasm and optic nerves

**Table 2:** Immunohistochemical profiles of spindle cell oncocytoma cases

Case	EMA	S100	Galectin-3	GFAP	Chromogranin	TTF-1	MIB-1
1	+	+	+	-	-	+	5%
2	+	+	+	-	-	+	2%
3A	-	+	+	-	-	+	5%
3B	+ (focal)	+	+	-	-	+	5%

We performed whole exome next-generation sequencing on each SCO case, using matched DNA as control. Sequencing revealed 43 nonsynonymous somatic mutations, insertions, or deletions (Table 3). Among the mutations present, samples 3A and 3B both showed a Q61R mutation in the Harvey rat sarcoma viral oncogene homolog (*HRAS*) gene on chromosome 11, a specific variant that has been previously reported in multiple cancers [10–13]. Cases 3A and 3B also both showed two frameshift mutations in the multiple endocrine neoplasia type 1 (*MEN1*) gene, also on chromosome 11; these variants have been previously reported as both germline and sporadic mutations in tumors of the pituitary and other sites [14].

**Table 3:** Mutations identified by whole exome sequencing of spindle cell oncocytoma cases

Gene	Chr.	Variant	Protein Change	Allele Frequency	Case	Functional Prediction Score (SIFT)
*NAV1*	1	Missense Mutation	L85V	0.31, 0.15	3A, 3B	0.00
*TCEB3*	1	Frameshift Deletion	K451fs	0.16	3A	—
*ADD2*	2	Missense Mutation	E480K	0.11	1	0.00
*C2orf16*	2	Nonsense Mutation	Q788*	0.39, 0.20	3A, 3B	—
*FZD7*	2	Missense Mutation	D3N	0.41, 0.27	3A, 3B	0.30
*SH3BP4*	2	Missense Mutation	D922G	0.14	1	0.00
*STAT4*	2	Missense Mutation	E388D	0.44, 0.25	3A, 3B	0.04
*ZNF717*	3	Frameshift Insertion	T45fs	0.4	2	—
*FAT1*	4	Missense Mutation	N109H	0.25	1	0.00
*FAT4*	4	Missense Mutation	N3706S	0.25	3B	0.10
*ANKH*	5	Missense Mutation	E43D	0.39, 0.09	3A, 3B	0.08
*ASCC3*	6	Missense Mutation	S221Y	0.12	3A	1.00
*EXOC2*	6	Missense Mutation	H736Q	0.33, 0.21	3A, 3B	0.34
*GPR115*	6	Missense Mutation	N230S	0.13	2	0.74
*GTPBP2*	6	Missense Mutation	R335Q	0.11	1	—
*PIK3CG*	7	Missense Mutation	E1073K	0.38, 0.15	3A, 3B	0.47
*SND1*	7	Missense Mutation	S578N	0.18	2	0.17
*TRPV5*	7	Missense Mutation	M440T	0.13	3A	0.25
*AGO2*	8	Missense Mutation	P430R	0.13	2	0.00
*C8orf76*	8	Frameshift Deletion	PERR21fs	0.47	3A	—
*TRPM6*	9	Missense Mutation	L333V	0.12, 0.14	3A, 3B	0.14
*CALHM1*	10	Missense Mutation	R178C	0.40, 0.13	3A, 3B	0.02
*CBL*	11	Missense Mutation	R280Q	0.41, 0.21	3A, 3B	0.01
*HRAS*	11	Missense Mutation	Q61R	0.42, 0.19	3A, 3B	0.04
*IPO7*	11	Nonsense Mutation	Y689*	0.21	3B	—
*MEN1*	11	Frameshift Deletion	K459fs	0.36, 0.17	3A, 3B	—
*MEN1*	11	Frameshift Deletion	LV117fs	0.36, 0.18	3A, 3B	—
*OR1S2*	11	Missense Mutation	P300S	0.42, 0.20	3A, 3B	0.00
*XRRA1*	11	Missense Mutation	R76Q	0.12	1	0.77
*CCER1*	12	Nonsense Mutation	R40*	0.13	2	—
*ITGA7*	12	Missense Mutation	D312A	0.42, 0.13	3A, 3B	0.43
*TAOK3*	12	Missense Mutation	E496D	0.39, 0.14	3A, 3B	0.11
*CCNK*	14	Missense Mutation	G53A	0.26	3B	0.27
*KLHDC1*	14	Frameshift Deletion	W28fs	0.36	1	—
*LTB4R*	14	Nonsense Mutation	Y172*	0.35, 0.14	3A, 3B	—
*RIN3*	14	Nonsense Mutation	W275*	0.06, 0.21	3A, 3B	—
*CHD2*	15	Missense Mutation	R550S	0.19	1	0.00
*DENND4A*	15	Missense Mutation	R865C	0.1	1	0.00
*LTK*	15	Missense Mutation	A480T	0.13	1	0.11
*TYRO3*	15	Missense Mutation	A48V	0.18	2	0.08
*SBK1*	16	Frameshift Deletion	G304fs	0.33	3B	—
*CD300A*	17	Missense Mutation	W49L	0.21	3B	0.00
*FMNL1*	17	Missense Mutation	P49R	0.27, 0.10	3A, 3B	0.00

Other tumor-associated genes found to be mutated as single events in individual tumors in the SCOs within our cohort include FAT atypical cadherin 1 (*FAT1*) [15], staphylococcal nuclease domain-containing protein 1 (*SND1*) [16, 17], Cbl proto-oncogene E3 ubiquitin protein ligase (*CBL*), frizzled class receptor 7 (*FZD7*), phosphatidylinositol-4,5-bisphosphate 3-kinase subunit gamma (*PIK3CG*), and SH3 domain binding kinase 1 (*SBK1*).

### Copy number profile of SCO

We found no significant recurrent copy number changes or aneuploidy across the examined cohort of SCO cases. We observed a loss of chromosome 13 in case 3A, which was not detected in the recurrent tumor, case 3B (Supplementary Figure S1). However, this may be attributable to a lower tumor cell fraction in case 3B, limiting its detection.

### Immunohistochemical assessment of MAPK and PI3K signaling pathways

**Figure 2:**
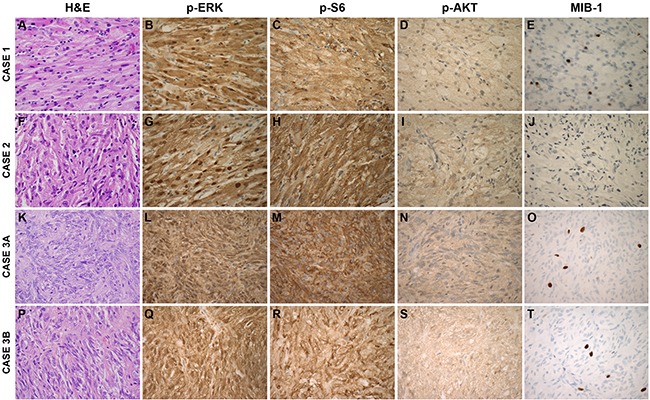
MAPK and PI3K Pathway Signaling in Spindle Cell Oncocytoma Cases Tissue sections were stained with H&E or immunohistochemistry for MIB-1, phosphorylated ERK (p-ERK), phosphorylated AKT (p-AKT), and phosphorylated S6 (p-S6) proteins. (600X magnification)

Prompted by the *HRAS* mutation identified in cases 3A and 3B, we examined activation of its canonical intracellular signaling cascade, the mitogen-activated protein kinase (MAPK) pathway. Ras signals activate Raf, resulting in phosphorylation of downstream MEK and of ERK. This leads to multiple cellular responses, including phosphorylation of ribosomal protein S6, which regulates protein translation and activates cell cycle regulators. We found robust expression (>90% positivity) of downstream pathway effectors, phosphorylated ERK (p-ERK) and S6 (p-S6), in all four SCO cases, using immunohistochemistry (Figure 2). In contrast, IHC for phosphorylated protein kinase B (p-AKT) showed only a weak signal, indicating basal activation of the phosphoinositide 3-kinase (PI3K) pathway.

## DISCUSSION

Strong evidence of activated downstream effectors of the MAPK pathway in each pituitary SCO tumor in this study suggests a perturbation that may drive cellular proliferation. In cases 3A and 3B, we identified an *HRAS* Q61R mutation by whole exome sequencing, which is associated with multiple other cancers and may have caused MAPK pathway activation. Case 2 contained a mutation in *SND1*, which has been reported to be involved in glioblastoma and carcinomas of the colon, prostate, and liver [16, 18–20]. *SND1* is a component of the RNA-induced silencing complex (RISC) and has been reported to activate the MAP kinase ERK [17]. Case 1 contained a mutation in the tumor suppressor *FAT1* atypical cadherin gene, which has been implicated in glioblastoma, colorectal adenocarcinoma, and head and neck squamous cell carcinoma [15]. While FAT1 is best known for promoting Wnt signaling, FAT1 expression has also been associated with ERK activation [21]. Therefore, mutations in *HRAS*, *SND1*, and *FAT1* may constitute separate genetic drivers that underlie the common MAPK activation observed in each SCO.

While our immunohistochemical and exome sequencing findings point to MAPK pathway activation in SCOs, the finding of two *MEN1* mutations in cases 3A and 3B suggests that biallelic inactivation of *MEN1* may be a second mechanism underlying neoplasia in SCO. Inactivation of both *MEN1* alleles has been found in multiple endocrine tumors, including parathyroid adenoma, insulinoma, and a small subset of pituitary adenomas [22].

*HRAS* mutations have been previously associated with increased aggressiveness in pituitary adenomas [23, 24]. Given this, it is noteworthy that cases 3A and 3B, which displayed rapid recurrence leading to repeat resections, demonstrated a pathogenic *HRAS* mutation. As such, *HRAS* mutation may be an indicator of more aggressive behavior in SCO.

The recurrent tumor of case 3B may also be related to the acquisition of new somatic mutations not present in the initial tumor, case 3A. Newly mutated genes identified in case 3B include FAT atypical cadherin 4 (*FAT4*), Importin 7 (*IPO7*), Cyclin K (*CCNK*), SH3 domain-binding kinase 1 (*SBK1*), and *CD300A*. Of these, *FAT4*, *CCNK*, and *SBK1* have been previously linked to neoplasia [25–27] and may contribute to the aggressive behavior demonstrated by case 3.

Interestingly, the similarities in presentation between SCOs and pituitary adenomas are reflected in their genetic profiles as well. Various *MEN1* mutations have been implicated in pituitary adenoma [14], and, as mentioned earlier, pituitary adenomas with *HRAS* mutations show increased aggressiveness. The genetic similarity between SCO case 3 reported here and pituitary adenoma raises the question of diagnostic overlap. However, the immunohistochemical profile, including the absence of neuroendocrine markers and the presence of S100, strongly suggest that case 3 is indeed a spindle cell oncocytoma, rather than a pituitary adenoma [1, 28–30].

Pituitary adenomas have been associated with mutations in numerous other genes, including succinate dehydrogenase (*SDH*) [31], ubiquitin-specific peptidase 8 (*USP8*) [32, 33], cyclin-dependent kinase inhibitor 1B (*CDKN1B*) [34], aryl hydrocarbon receptor interacting protein (*AIP*) [35], and cAMP-dependent protein kinase type 1-alpha regulatory subunit (*PRKAR1A*) [36]. These mutations were not identified in our whole exome sequences of spindle cell oncocytoma. Cytogenetic studies of pituitary adenoma have shown scattered chromosome gains and losses, without a significant recurrent signature [37, 38]. The minimal chromosomal abnormalities we observed in our SCOs are consistent with the copy number profiles of some non-functional pituitary adenomas.

Scarce genetic information on pituicytoma is available for comparison with SCO. Comparative genomic hybridization (CGH) performed on one case of pituicytoma showed multiple copy number imbalances, with losses on 1p, 14q, and 22q and a gain on 5p [39]. This pattern appears distinct from our findings for SCO. Overall, we did not identify any significant copy number profile changes in SCO that have been reported in pituitary adenoma or pituicytoma.

In this report, we present four cases of SCO, using whole exome sequencing to reveal abnormal MAPK pathway signaling, suggesting it may be a common mechanism underlying oncogenesis as a shared phenotypic endpoint of various driver mutations. Inhibition of MAPK pathway signal transducers, or downstream nodes such as MEK, is under active clinical investigation in multiple other cancers [40–42]. Consequently, targeted inhibition of MAPK pathway signaling may offer an opportunity for treatment of spindle cell oncocytomas that cannot be controlled by surgical resection alone. Mutational profiling of many other tumor types has opened up successful personalized targeted medical treatments, and our findings suggest spindle cell oncocytomas may also be amenable to this approach.

## MATERIALS AND METHODS

### Sample selection

Analysis of data generated from tumor specimens and clinical information was conducted under a Dana-Farber/Brigham and Women's Cancer Center (DF/BWCC) Institutional Review Board (IRB)-approved protocol. Histologic diagnosis was confirmed on all samples by a board-certified neuropathologist (S.H.R.) and representative paraffin-embedded tissue with average estimated purity >70% was selected. Tumor DNA was extracted from 1 mm cores and normal DNA was prepared from patient salivary samples using standard techniques (Oragene kit, DNA Genotek, Kanata, Ontario, Canada; and Qiagen, Valencia, CA). The tumor-normal pairs were confirmed by mass spectrometric genotyping with an established 48-SNP panel (Sequenom, San Diego, CA) [43].

### Whole exome sequencing, mutation analysis, and copy number analysis

Whole exome sequencing was performed as previously described [44]. DNA was sonicated to 150 bp fragments, size selected with Agencourt AMPure XP beads, and ligated to specific barcoded adapors (Illumina TruSeq; Illumina Inc., San Diego, CA) for multiplexed analysis. Exome hybrid capture was performed using the Agilent SureSelect hybrid capture kit (Whole Exome v4; Agilent Technologies, Santa Clara, CA) and sequenced on a HiSeq 2500 system (Illumina Inc., San Diego, CA). All samples achieved at least 80X depth of coverage across exons.

Read pairs were aligned to the hg19 reference sequence using the Burrows-Wheeler Aligner [45], and sample reads sorted and duplicate-marked using SAMtools and Picard. Bias in base quality score assignments due to flowcell, lane, dinucleotide context, and machine cycle were analyzed and recalibrated, and local realignment around insertions or deletions (indels) was achieved using the Genome Analysis Toolkit (GATK) [46, 47].

Somatic mutations and short indels were called and post-filtered using MuTect [48] and IndelLocator [49, 50]. These were annotated to genes and compared to events in the Catalogue of Somatic Mutations in Cancer (COSMIC) using Oncotator and also manually verified in the sequence output through visualization in the Integrated Genome Viewer (IGV). To analyze somatic copy number alterations from whole exome data, we used an allelic copy number pipeline, consisting of the ReCapSeg, Allelic Capseg and ABSOLUTE algorithms. ReCapseg detects total copy ratios from whole-exome sequencing data and performs a tangent normalization against a panel of normal exomes. Allelic capseg takes the output of ReCapseg and splits total copy ratios into homologue-specific copy ratios (HSCRs) from segmental estimates of multipotent allelic copy-ratios at heterozygous loci incorporating the statistical phasing software (BEAGLE) and population haplotype panels (HAPMAP3) [51–53]. Allele-specific somatic copy number alterations and tumor ploidy status were assessed with the ABSOLUTE algorithm [53].

Prediction of possible functional effect of the identified mutations was performed using the SIFT (Sorting Intolerant from Tolerant) Human Protein algorithm (J. Craig Venter Institute) [54]. The SIFT prediction score ranges from 0 to 1, and is the scaled probability of an amino acid substitution being tolerated. Amino acid substitutions with scores that fall below 0.05 are predicted to affect protein function. Notably, such prediction algorithms may be more useful for loss of function of tumor suppressor genes than for predicting gain of function for proto-oncogenes.

### Immunohistochemistry

Diaminobenzidine (DAB) brightfield staining was performed according to standard protocols on 5 μm paraffin sections [55]. Antigens were retrieved using heat and 10 mM sodium citrate buffer (pH 6.0). The following primary antibodies were utilized: S100 (DAKO, 1:1000 dilution), vimentin (DAKO, 1:400), EMA (DAKO, 1:200), galectin-3 (Fitzgerald Industries, 1:100), chromogranin (Thermo Scientific, 1:4000), GFAP (DAKO, 1:2,000), TTF-1 (DAKO, 1:300), CD68 (DAKO, 1:1000), p-ERK (Cell Signaling, 1:200), p-AKT (Cell Signaling, 1:50), p-S6 (Cell Signaling, 1:50), and MIB-1 (Ki-67) (Leica, 1:200). Counterstaining for nuclei was performed using Mayer's hematoxylin stain, and cover slips were mounted using Permount (Fisher Scientific).

## SUPPLEMENTARY FIGURE


