# Supplementary material for: MAPK activation and HRAS mutation identified in pituitary spindle cell oncocytoma
Source: Oncotarget. 2016 May 9;7(24):37054–63. doi: 10.18632/oncotarget.9244 (PMC5095058; doi:10.18632/oncotarget.9244)
Supplement: Supplementary file 1 [file oncotarget-07-37054-s001.pdf]

SUPPLEMENTARY FIGURE

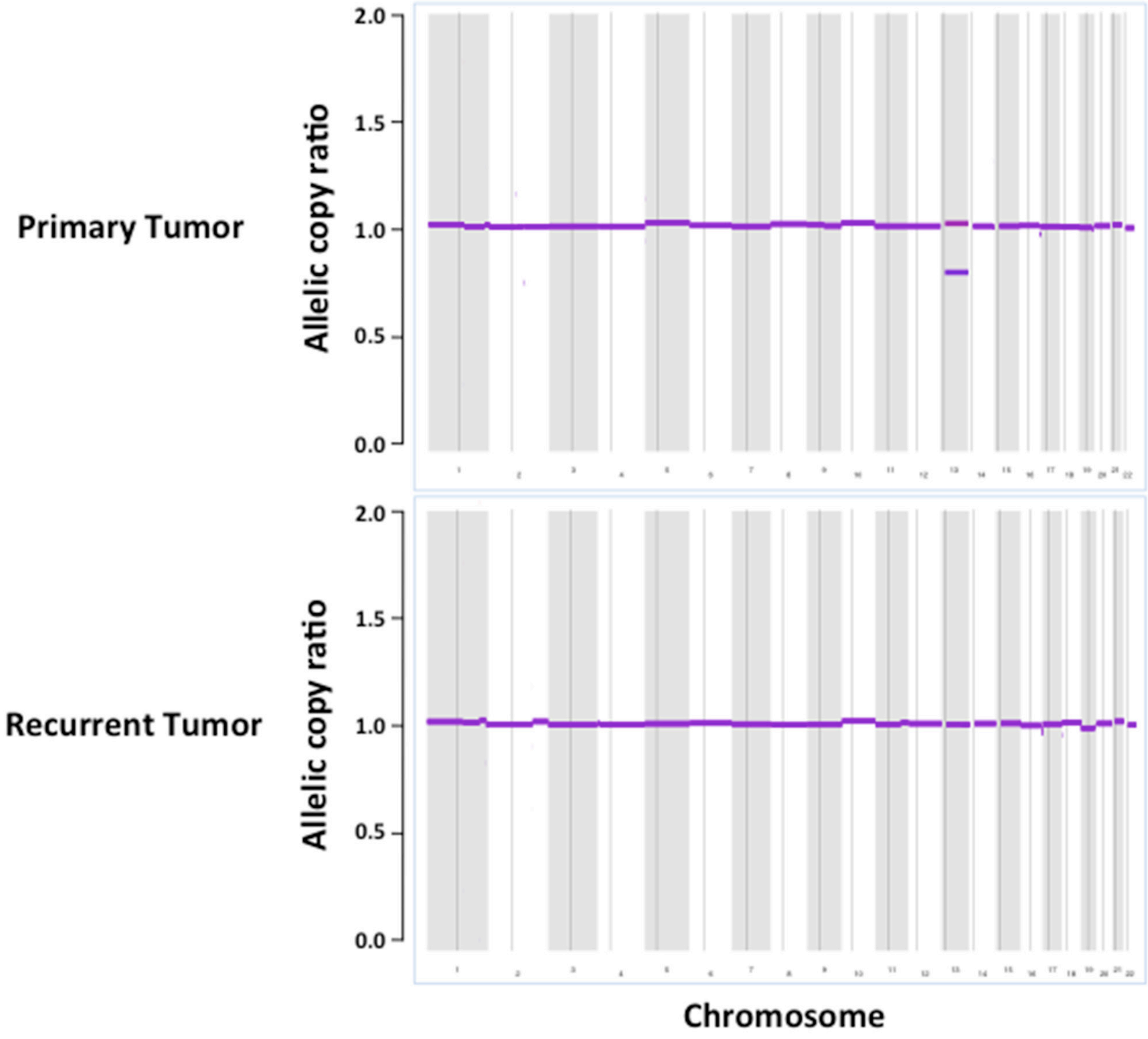

Supplementary Figure S1: Chromosomal Copy Number Profile of a Recurrent Spindle Cell Oncocytoma. Allelic copy number ratios are shown by chromosome for the primary tumor (case 3A) and the recurrent/residual tumor (case 3B).
